# Development and Characterization of Novel LipoCEST Agents Based on Thermosensitive Liposomes

**DOI:** 10.2463/mrms.mp.2015-0039

**Published:** 2016-02-03

**Authors:** Shuki MARUYAMA, Junpei UEDA, Atsuomi KIMURA, Kenya MURASE

**Affiliations:** Department of Medical Physics and Engineering, Division of Medical Technology and Science, Faculty of Health Science, Graduate School of Medicine, Osaka University 1-7 Yamadaoka, Suita, Osaka 565-0871, Japan

**Keywords:** lipoCEST, thermosensitive liposomes, theranostics, hyperthermia

## Abstract

**Purpose:**

To develop a novel probe for chemical exchange saturation transfer magnetic resonance imaging (CEST MRI) based on thermosensitive liposomes (lipoCEST) for theranostics, in which diagnostics and therapy are integrated into a single platform.

**Methods:**

We developed two kinds of lipoCEST agents. The first kind encapsulated dysprosium (Dy)-1,4,7,10-tetraazacyclododecane-1,4,7,10-tetraacetic acid (DOTA)-Na·3NaCl, terbium-DOTA-Na·3NaCl, or thulium-DOTA-Na·3NaCl into the inner cavity of thermosensitive liposomes, while the second kind encapsulated Dy-DOTA-Na and incorporated amphiphilic metal complex [thulium-diethylenetriamine pentaacetic acid-bis (stearylamide) (Tm-DTPA-BSA)] as a membrane constituent. The nuclear magnetic resonance (NMR)- and Z-spectra of these lipoCEST agents were acquired at various temperatures on a 9.4T MRI scanner. To investigate their applicability to the drug release induced by hyperthermia, we also encapsulated a fluorescent dye (calcein) into the inner cavity of liposomes and measured calcein release after warming them.

**Results:**

The intra- and extraliposomal water signals could be differentiated in all agents from their NMR- and Z-spectra. The agent incorporating Tm-DTPA-BSA showed the largest chemical shift (approximately 15 ppm) derived from the intraliposomal water protons. The calcein retained in this agent was successfully released at 44°C. The agent incorporating 30 mol% of Tm-DTPA-BSA in its membrane released more calcein at 42–44°C than that of the agent incorporating 10 mol%.

**Conclusion:**

We developed novel thermosensitive lipoCEST agents and characterized them. Our preliminary results suggest that they are useful and can be applied to theranostics.

## Introduction

Magnetic resonance imaging (MRI) is a valuable diagnostic tool providing anatomical and functional information. Recently, chemical exchange saturation transfer (CEST) MRI has emerged as a new molecular imaging technique that can indirectly image exchangeable solute protons resonating at different frequencies from those of bulk water protons.^1^ The CEST MRI has attracted increasing attention, because it can offer an effective method to acquire the *in vivo* physiological information such as pH^2^ that the conventional MRI cannot obtain easily.

The reagent, called the CEST agent, was first introduced by Wolff et al.^3–5^ and is mainly classified into diamagnetic CEST (diaCEST), paramagnetic CEST (paraCEST), and liposomal CEST (lipoCEST) agents. While efforts to develop novel diaCEST and paraCEST agents have continued, attempts have also been made to develop lipoCEST agents that exploit nano-carrier systems because their sensitivity is higher than that of the others.^6,7^ The lipoCEST agent composed of liposomes encapsulating the shift reagent is especially anticipated to be a powerful CEST agent for the purpose of the therapy integrated with diagnosis, so-called “theranostics,” because it can also encapsulate drugs.

A lipoCEST agent encapsulating the exogenous shift reagent in the inner cavity can shift the resonating frequency of intraliposomal water protons by 1 to 5 ppm from that of extraliposomal water protons.^7–9^ A lipoCEST agent having a large number of water protons in the inner cavity can increase the sensitivity.^7^ However, it is difficult to obtain a chemical shift between intra- and extraliposomal water protons (δ_intralipo_) larger than 4 ppm. Because a larger δ_intralipo_ is desirable to saturate intraliposomal water protons more efficiently and to reduce the interference with the magnetization transfer effect associated with endogenous proteins,^7^ the above problem should be addressed to apply the lipoCEST agent to *in vivo* molecular imaging.

The δ_intralipo_ is characteristically given by the sum of three contributions, i.e.,

$$\delta_{\text{intralipo}}=\delta_{\text{DIA}}+\delta_{\text{BMS}}+\delta_{\text{HYP}},$$

where δ_DIA_ is the diamagnetic contribution (negligible), δ_BMS_ is the bulk magnetic susceptibility (BMS) contribution, and δ_HYP_ is the hyperfine contribution.^7–9^ Because δ_BMS_ is almost zero for conventional spherical liposomes, δ_intralipo_ is only given by δ_HYP_ for these liposomes. In contrast, δ_BMS_ becomes non-zero for non-spherical liposomes due to its dependency on the orientation of liposomes to an external magnetic field, the concentration of the contents, and the effective magnetic moment of paramagnetic complex.^7–9^ Therefore, it is expected that a larger δ_intralipo_ can be obtained by further distorting the shape of liposomes. Terreno et al. have reported that non-spherical liposomes can be obtained by shrinking spherical liposomes through osmotic stress.^8–10^

In addition, the incorporation of paramagnetic lanthanide complex into the liposomal membrane can regulate the orientation of liposomes to a static magnetic field.^7–9^ In the present study, to validate and extend these studies, we prepared the lipoCEST agent incorporating amphiphilic metal complex in the liposomal membrane after distorting their shape. We noted that it is also important to regulate δ_intralipo_ for obtaining the images of CEST MRI without artifacts induced when δ_intralipo_ is close to zero.^11^ Therefore, we attempted to use a combination of two lanthanide (Ln) complexes, Ln-DOTA (DOTA: 1,4,7,10-tetraazacyclododecane-1,4,7,10-tetraacetic acid) and Ln-DTPA-bis (stearylamide) (DTPA: diethylenetriamine pentaacetic acid), because these reagents are capable of widely changing the δ_intralipo_.

If we were to use thermosensitive liposomes as the base for the lipoCEST agent, we expected that theranostics could be easily accomplished, integrating the diagnosis using MRI and the therapy using drug release induced by hyperthermia into a single platform. Recently, Tagami et al. have reported a novel thermosensitive liposome formulation composing of 1,2-dipalmitoyl-sn-glycero-3-phosphocholine (DPPC) and polyoxyethylene (20) stearyl ether (Brij78^®^) at a molar ratio of 96:4 (hyperthermia activated cytotoxic (HaT)-liposome), and that HaT-liposome encapsulating gadolinium (Gd)-DTPA can be applied to hyperthermia combined with drug delivery monitoring by MRI.^12^ The HaT-liposome appears to be useful for preparing the lipoCEST agent, because it is simple to synthesize and has sufficient thermosensitivity.^13^ To the best of our knowledge, however, there are no reports of surfactants such as Brij78 being used in lipoCEST agent. Therefore, we also investigated the usefulness of these surfactants in the preparation of the lipoCEST agents.

## Materials and Methods

### Materials

DPPC and polyoxyethylene (2) stearyl ether (Brij72^®^) were purchased from Wako Pure Chemical Industries, Ltd. (Osaka, Japan). Bis [N,N-bis (carboxymethyl) aminomethyl] fluorescein (calcein) was manufactured by Dojindo Co., Ltd. (Kumamoto, Japan) and purchased from Wako Pure Chemical Industries. DOTA was purchased from Macrocyclics, Inc. (Dallas, TX, USA). Dysprosium chloride hexahydrate (DyCl_3_·6H_2_O) was purchased from Sigma-Aldrich Japan Co. (Tokyo, Japan). Terbium chloride hexahydrate (TbCl_3_·6H_2_O) and thulium chloride hexahydrate (TmCl_3_·6H_2_O) were purchased from Tokyo Chemical Industry Co., Ltd. (Tokyo, Japan). DTPA-bis (stearylamide) (gadolinium salt) was obtained from Avanti Polar Lipids, Inc. (Alabaster, AL, USA).

### Synthesis of Tm-DTPA-bis (stearylamide)

Tm-DTPA-bis (stearylamide) (Tm-DTPA-BSA) was synthesized from Gd-DTPA-BSA and thulium chloride (TmCl_3_·6H_2_O). First, 300 mg of Gd-DTPA-BSA was dissolved in 25 mL of ethanol, and the solution was heated to 80°C. A 10 mL aqueous solution of TmCl_3_·6H_2_O was dropped into the ethanol solution of Gd-DTPA-BSA and stirred for 30 min. The solution was slowly evaporated for 1 h and distilled water was added to the solution until the complex was perfectly crystallized. The resultant solution was kept at room temperature overnight.

### Preparation of thermosensitive lipoCEST agents

The thermosensitive lipoCEST agent was prepared according to the procedure published by Terreno et al.^11^ First, 20 mg of DPPC and Brij72 were mixed at a molar ratio of 90:10 and dissolved in ethanol at room temperature. The solvent was removed by a rotary evaporator until a thin film was formed in a round-bottom flask. Then, it was dried for 24 h. The thin film was hydrated at 65°C with 1 mL of aqueous solution containing Ln-DOTA with pH being adjusted at 7.4. The solution was sonicated for 5 min, and the sonication was repeated 4 times with an interval of 1 min. Then, it was extruded at 65°C. The suspension was dialyzed against isotonic NaCl aqueous solution at 4°C in order to remove the unencapsulated shift reagent. Non-spherical liposomes were prepared by dialyzing the suspension against hypertonic NaCl aqueous solution and by adding the osmotic stress. The size of liposome that influences the exchange rate of protons in water through the membrane^14^ was determined by dynamic light scattering (SALD-7100, Shimadzu Co., Kyoto, Japan) (about 70 nm, data not shown). We prepared two kinds of lipoCEST agents (denoted by lipoA and lipoB) as follows: lipoA composed of DPPC and Brij72 at a molar ratio of 90:10 and lipoB composed of DPPC and Brij72 at a molar ratio of 90:10 and incorporating 10 or 30 mol% of Tm-DTPA-BSA.

### NMR experiments

All nuclear magnetic resonance (NMR) spectral measurements were performed on an Agilent Unity INOVA 400WB high-resolution spectrometer (9.4T) equipped with VNMR 6.1C software (Agilent Technologies Inc., Santa Clara, CA, USA). Acquisition parameters for all NMR spectral measurements were as follows: spectral bandwidth = 50 kHz; data complex points = 30 k; relaxation delay = 41 s; number of acquisitions = 2; and flip angle = 75°. The NMR experiments were performed under three conditions. First, the NMR spectra of the lipoA agents encapsulating 80 mM Dy-DOTA-Na·3NaCl, Tb-DOTA-Na·3NaCl, and Tm-DOTA-Na·3NaCl were acquired at 36, 38, 40, and 42°C. Second, the NMR spectra of the lipoA agents encapsulating 20 mM Dy-DOTA-Na·3NaCl, Tb-DOTA-Na·3NaCl, or Tm-DOTA-Na·3NaCl dialyzed against NaCl aqueous solution with various osmotic pressures [isotonic (170), 300, 600, 900, and 1200 mOsm] were acquired at 36°C. The NMR spectra of the lipoA agents encapsulating 20 mM Dy-DOTA-Na·3NaCl or Tm-DOTA-Na·3NaCl and dialyzed against NaCl aqueous solution with an osmotic pressure of 1200 mOsm were also acquired at 36, 38, 40, and 42°C. Finally, the NMR spectra of the lipoB agents encapsulating 20 mM Dy-DOTA-Na with an osmotic pressure of 40 mOsm dialyzed against NaCl solution with an osmotic pressure of 300 mOsm were acquired at 36, 38, 40, 42, and 44°C. In these NMR measurements, the overall time per NMR spectrum was 82 s at each temperature and a pre-delay time of at least 5 min was set for each temperature level to ensure that the temperature of the sample became stable.

The Z-spectra of the lipoB agents were acquired at 38°C. They were also acquired at 44°C after the liposomal suspension was incubated for 5 min. The Z-spectrum is defined as the NMR signal from bulk water protons acquired with off-resonant saturation normalized by that acquired with on-resonant saturation.^15^ The Z-spectrum was acquired with a frequency offset ranging from −23 ppm to 23 ppm with an interval of 1 ppm. The saturation was performed by a continuous wave (CW) radiofrequency (RF) irradiation mode. The saturation power and irradiation time were taken as 2.1 μT and 1 s, respectively. The parameters of the Z-spectral measurements were the same as those of the NMR spectral measurements, except for the flip angle (= 20°), the number of acquisitions (= 1), and the relaxation delay of 6 s. The overall scan duration to acquire 47 NMR spectra in a Z-spectrum was 276 s.

### Preparation of thermosensitive lipoCEST agent loading calcein

The thermosensitive lipoCEST agent loading calcein were prepared according to the procedure published by Pradhan et al.^16^ For the preparation of the agent loading calcein, 20 mg/mL of the lipid mixture composing of DPPC and Brij72 at a molar ratio of 90:10 and incorporating 10 or 30 mol% of Tm-DTPA-BSA was dissolved in ethanol in a round-bottom flask. The solvent was removed by a rotary evaporator and desiccated for 1 day. The lipid mixture was hydrated at 65°C by adding a phosphate buffered saline (PBS) solution of calcein (63 mM) with the pH adjusted at 7.4. The suspension obtained was sonicated for 5 min, and the sonication was repeated four times with an interval of 1 min. To remove unencapsulated calcein, gel chromatography was carried out using Sepharose gel in a CL-2B column (2 cm in diameter and 50 cm in length) (GE Healthcare Japan Co. Ltd., Tokyo, Japan) with a flow rate of 1 mL/min, and PBS was used as the elution solution.

To prepare non-spherical liposomes, the suspension was dialyzed against hypertonic NaCl solution with various osmotic pressures as in the preparation of the thermosensitive lipoCEST agents without loading calcein.

### Temperature-dependent release of calcein from thermosensitive lipoCEST agent

The temperature-dependent release of calcein from thermosensitive lipoCEST agents was measured using the self-quenching phenomenon of calcein fluorescence at high concentration that is lost by the release.^16^ Briefly, 60 μL suspension of the lipoCEST agent containing calcein was added to 600 μL PBS in a micro tube. The samples obtained from the suspension were incubated for 20 min at each temperature level starting from 26°C and increasing to 34, 36, 38, 40, 42, and 44°C. Twenty microliters of each sample was diluted by adding 2 mL of PBS, and the sample was placed in a 96-well plate for the measurement of fluorescence intensity. The fluorescence intensity was measured using a plate reader (F-7000, Hitachi Co. Ltd., Tokyo, Japan) at an excitation wavelength of 485 nm and an emission wavelength of 520 nm. To measure the maximum release of calcein, 60 μL of the suspension was added to 600 μL PBS containing 1% Trition X-100 (Wako Pure Chemical Industries Ltd., Osaka, Japan) at a sufficient weight ratio (6 μL) to destroy the liposomal membrane. The percentage of calcein release from thermosensitive liposomes was calculated as follows:

[1] $$\text{Calcein}\;\text{release}\;(\%)=\frac{F_{f}-F_{i}}{F_{t}-F_{i}}\times 100,$$

where *F_i_* and *F_f_* denote the initial and final fluorescence intensities of the liposome suspension at a given temperature, respectively, and *F_t_* is the fluorescence intensity of Triton X-100 treated samples.

### Statistical analysis

All data are expressed as the mean ± standard error (SE). For comparison among groups with various mol% of Tm-DTPA-BSA (0, 10, and 30 mol%), one-way analysis of variance (ANOVA) was used. Statistical significance was determined by Tukey-Kramer multiple comparison test. For comparison between two groups with different mol% of Tm-DTPA-BSA, the Student’s *t*-test was used. A *P* value less than 0.05 was considered statistically significant. All analyses were performed using Excel 2010 (Microsoft Co., Redmond, WA, USA) and Statcel3 software (OMS Publishing Inc., Saitama, Japan).

## Results

### NMR measurements

**Fig. 1.**
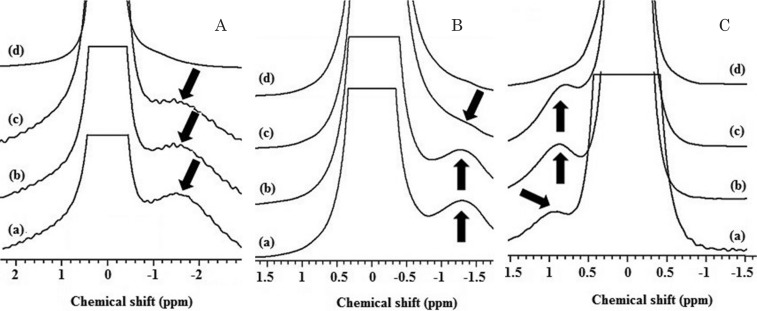
Nuclear magnetic resonance (NMR) spectra obtained from the lipoA agent encapsulating 80 mM dysprosium (Dy)-1,4,7,10-tetraazacyclododecane-1,4,7,10-tetraacetic acid (DOTA)-Na·3NaCl (**A**), terbium (Tb)-DOTA-Na·3NaCl (**B**), and thulium (Tm)-DOTA-Na·3NaCl (**C**), where lipoA composed of 1,2-dipalmitoyl-sn-glycero-3-phosphocholine (DPPC) and Brij72 at a molar ratio of 90:10 was dialyzed isotonic NaCl solution (680 mOsm). Labels a–d show the spectra measured at 36, 38, 40, and 42°C, respectively.

Figure 1 shows the NMR spectra obtained from the lipoA agents encapsulating Dy-DOTA-Na·3NaCl (A), Tb-DOTA-Na·3NaCl (B), and Tm-DOTA-Na·3NaCl (C) at various temperatures. Labels a–d in each graph show cases when the temperature was 36, 38, 40, and 42°C, respectively. The NMR spectral peaks derived from the intraliposomal water protons (shown by arrows) for Dy-DOTA-Na·3NaCl, Tb-DOTA-Na·3NaCl, and Tm-DOTA-Na·3NaCl were shifted by −2.0 ppm, −1.2 ppm, and +1.0 ppm, respectively, compared to bulk water. They disappeared at 42°C [(d) in each graph].

**Fig. 2.**
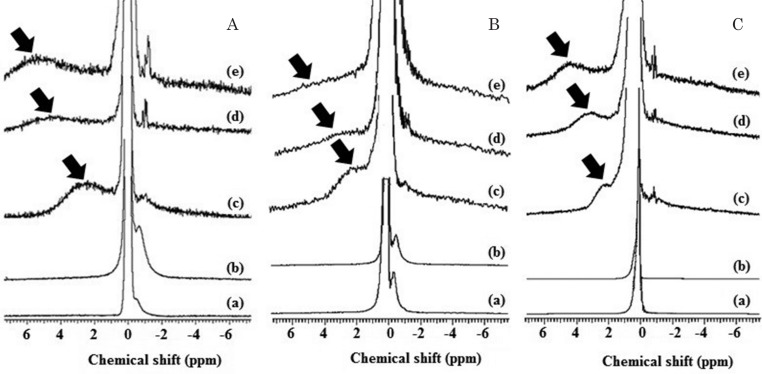
Nuclear magnetic resonance (NMR) spectra obtained from the lipoA agent encapsulating 20 mM dysprosium (Dy)-1,4,7,10-tetraazacyclododecane-1,4,7,10-tetraacetic acid (DOTA)-Na·3NaCl (**A**), terbium (Tb)-DOTA-Na·3NaCl (**B**), and thulium (Tm)-DOTA-Na·3NaCl (**C**) at 36°C. Labels a–e show cases when the osmotic pressure for dialysis against NaCl aqueous solution was isotonic (170), 300, 600, 900, and 1200 mOsm, respectively.

Figure 2 shows the NMR spectra obtained from the lipoA agents encapsulating Dy-DOTA-Na·3NaCl (A), Tb-DOTA-Na·3NaCl (B), and Tm-DOTA-Na·3NaCl (C) at 36°C. Labels a–e in each graph show cases when the osmotic pressure was isotonic (170), 300, 600, 900, and 1200 mOsm, respectively. As shown in Fig. 2, the chemical shift between the intra- (shown by arrows) and extraliposomal water protons increased with increasing osmotic pressure, suggesting that the lipoCEST agent exposed to hypertonic solution was shrunken with increasing osmotic pressure.

**Fig. 3.**
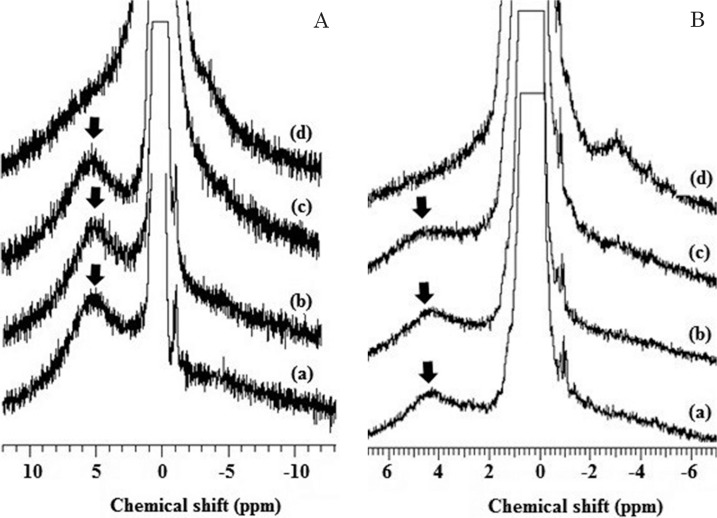
Nuclear magnetic resonance (NMR) spectra obtained from the lipoA agent encapsulating 20 mM dysprosium (Dy)-1,4,7,10-tetraazacyclododecane-1,4,7,10-tetraacetic acid (DOTA)-Na·3NaCl (**A**) and thulium (Tm)-DOTA-Na·3NaCl (**B**). Labels a–d shows cases when temperature was 36, 38, 40, and 42°C, respectively. Note that samples were dialyzed against NaCl solution with an osmotic pressure of 1200 mOsm.

Figure 3 shows the NMR spectra obtained from the lipoA agents encapsulating Tm-DOTA-Na·3NaCl (A) and Dy-DOTA-Na·3NaCl (B) at various temperatures. Labels a–d in each graph show cases when the temperature was 36, 38, 40, and 42°C, respectively. The NMR spectral peaks derived from the intraliposomal water protons (shown by arrows) disappeared at 42°C [(d) in each graph] in both agents.

**Fig. 4.**
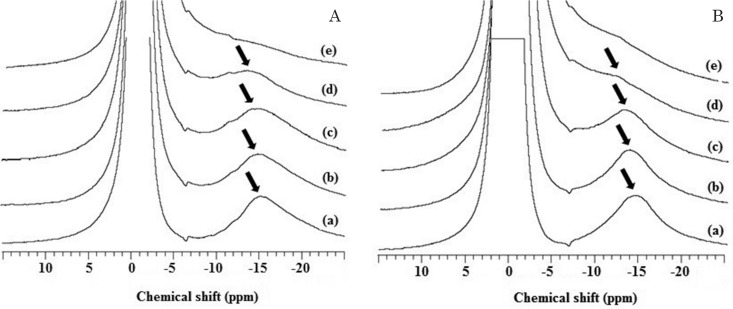
Nuclear magnetic resonance (NMR) spectra obtained from the lipoB agent encapsulating 20 mM dysprosium (Dy)-1,4,7,10-tetraazacyclododecane-1,4,7,10-tetraacetic acid (DOTA)-Na whose membrane includes 10 mol% (**A**) and 30 mol% of thulium-diethylenetriamine pentaacetic acid-bis (stearylamide) (Tm-DTPA-BSA) (**B**), where lipoB composes of 1,2-dipalmitoyl-sn-glycero-3-phosphocholine (DPPC) and Brij72 at a molar ratio of 90:10 and incorporates Tm-DTPA-BSA, and was dialyzed against NaCl solution with an osmotic pressure of 300 mOsm. Labels a–e show cases when the temperature was 36, 38, 40, 42, and 44°C, respectively.

Figure 4 shows the NMR spectra obtained from the lipoB agents encapsulating Dy-DOTA-Na with an osmotic pressure of 40 mOsm and incorporating 10 mol% (A) and 30 mol% of Tm-DTPA-BSA as membrane constituents (B) which were dialyzed against NaCl solution with an osmotic pressure of 300 mOsm. Labels a–e in each graph show cases when the temperature was 36, 38, 40, 42, and 44°C, respectively. As can be seen by comparing Figs. 3 and 4, the use of Dy-DOTA-Na instead of Dy-DOTA-Na·3NaCl as a shift reagent and the incorporation of Tm-DTPA-BSA into the liposomal membrane increased the chemical shift and reversed the direction of the chemical shift. Furthermore, the NMR spectral peaks derived from the intraliposomal water protons (shown by arrows) disappeared at 44°C [(e) in each graph] in both cases. Thus, the temperature at which the NMR spectral peak disappeared (44°C) was higher than was the case without Tm-DTPA-BSA (42°C) (Fig. 3).

**Fig. 5.**
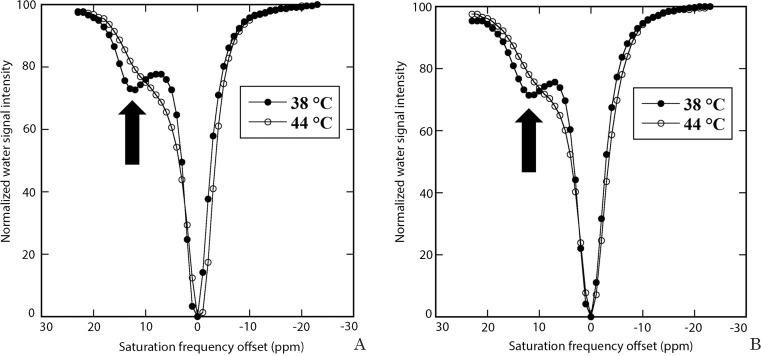
Z-spectra obtained from the lipoB agent encapsulating 20 mM dysprosium (Dy)-1,4,7,10-tetraazacyclododecane- 1,4,7,10-tetraacetic acid (DOTA)-Na whose membrane includes 10 mol% (**A**) and 30 mol% of thulium-diethylenetriamine pentaacetic acid-bis (stearylamide) (Tm-DTPA-BSA) (**B**), and was dialyzed against NaCl solution with an osmotic pressure of 300 mOsm. Closed and open circles show cases when the temperature was 38°C and 44°C, respectively. In these cases, the saturation power and irradiation time were taken as 2.1 μT and 1 s, respectively.

Figure 5 shows the Z-spectra obtained from the lipoB agents encapsulating Dy-DOTA-Na with an osmotic pressure of 40 mOsm and incorporating 10 mol% (A) and 30 mol% of Tm-DTPA-BSA as membrane constituents (B) which were dialyzed against NaCl solution with an osmotic pressure of 300 mOsm. The closed and open circles in each plot show the Z-spectra measured at 38°C and 44°C, respectively. As shown in Fig. 5, the CEST peaks (shown by arrows) appeared at approximately 15 ppm from that of bulk water in both cases at 38°C, and they almost disappeared at 44°C. The amount of Tm-DTPA-BSA did not significantly affect the position at which the CEST peak appeared.

### Calcein release measurements

**Fig. 6.**
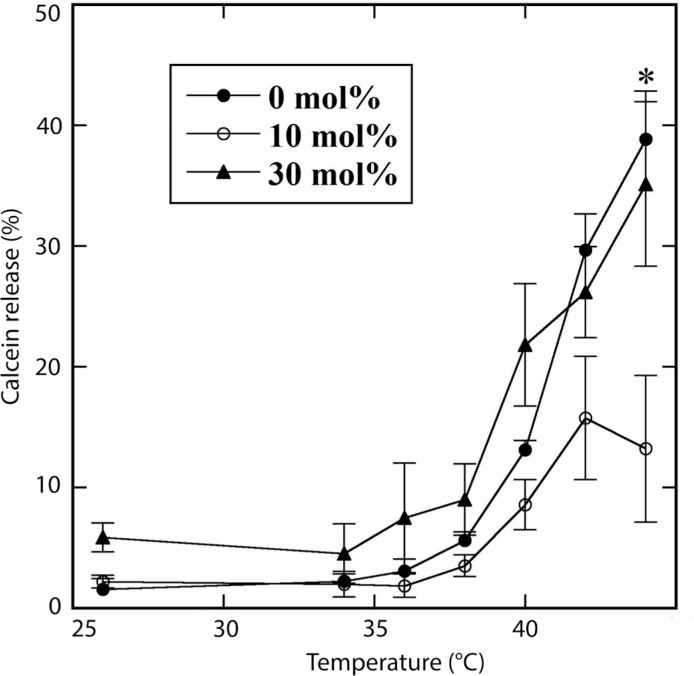
Relationship between temperature and the calcein release from the lipoB agent (closed circles) and the lipoB agents incorporating 10 mol% (open circles) and 30 mol% of thulium-diethylenetriamine pentaacetic acid-bis (stearylamide) (Tm-DTPA-BSA) (closed triangles) after incubation for 20 min. Data are represented by mean ± standard error (SE) for n = 4. *P < 0.05 for comparison between the lipoB agent and the lipoB agent incorporating 10 mol% of Tm-DTPA-BSA by Student’s *t*-test. Note that there were no significant differences among groups when analyzing by Tukey-Kramer multiple comparison tests.

In order to investigate the influence of Tm-DTPA-BSA on the thermosensitivity of the lipoCEST agent, the temperature dependency of the calcein release from the lipoB agents was measured after incubation for 20 min. Figure 6 shows the relationships between temperature and the calcein release calculated from Eq. [1] for cases when the amount of Tm-DTPA-BSA incorporated in the lipoB agent was 0 (closed circles), 10 (open circles), and 30 mol% (closed triangles). Although there were no significant differences among groups when analyzing by Tukey-Kramer multiple comparison test, there was a significant difference between the lipoCEST agent without Tm-DTPA-BSA and that incorporating 10 mol% of Tm-DTPA-BSA at 44°C when analyzing by Student’s *t*-test.

**Fig. 7.**
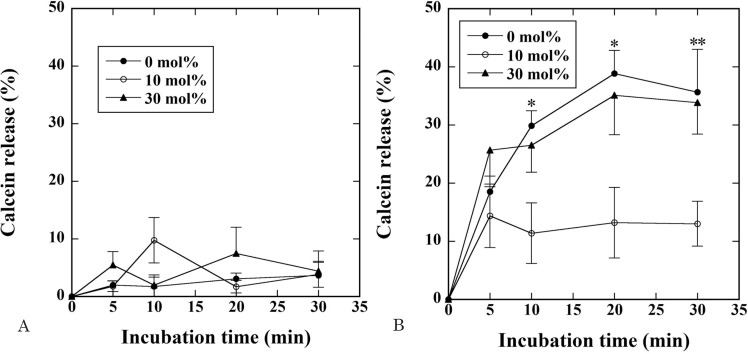
Calcein release from the lipoB agent (closed circles) and the lipoB agents incorporating 10 mol% (open circles) and 30 mol% of thulium-diethylenetriamine pentaacetic acid-bis (stearylamide) (Tm-DTPA-BSA) (closed triangles) as a function of incubation time. (**A**) and (**B**) show cases when the temperature was 36°C and 44°C, respectively. Data are represented by mean ± standard error (SE) for n = 4. *P < 0.05 for comparison between the lipoA agent and the lipoB agent incorporating 10 mol% of Tm-DTPA-BSA by Student’s *t*-test; **P < 0.05 for comparison between the lipoB agents incorporating 10 mol% and 30 mol% of Tm-DTPA-BSA by Student’s *t*-test. Note that there were no significant differences among groups when analyzing by Tukey-Kramer multiple comparison tests.

Figure 7 shows the calcein release calculated from Eq. [1] as a function of incubation time at 36°C (A) and 44°C (B) for cases when the amount of Tm-DTPA-BSA incorporated in the lipoB agent was 0 (closed circles), 10 (open circles), and 30 mol% (closed triangles). Although there were no significant differences among groups when analyzing by Tukey-Kramer multiple comparison test, there were significant differences between the lipoCEST agent without Tm-DTPA-BSA and that incorporating 10 mol% of Tm-DTPA-BSA for incubation times of 10 and 20 min and between the lipoCEST agents incorporating 10 mol% and 30 mol% of Tm-DTPA-BSA for an incubation time of 30 min when analyzing by Student’s *t*-test (Fig. 7B).

## Discussion

Future theranostic approaches may be based on nano- or microparticles like liposomes encapsulating contrast agents for imaging and drugs that can be released for targeted local drug delivery by external stimuli. It is well known that liposomes are biocompatible and biodegradable nanovesicles.^8–10^ Thermosensitive liposomes are one of the most attractive nanocarriers for theranostics, because they can rapidly release encapsulated drugs through the disruption of liposomal membranes by raising the temperature moderately.^13,17,18^ For this reason, some attempts have been made to apply thermosensitive liposomes to CEST MRI.^19^ Langereis et al. have developed a novel temperature-sensitive liposomal MRI contrast agent co-encapsulating [Tm(hpdo3a)(H_2_O)] and fluorinated compound, which allows drug carrier localization using ^1^H CEST MRI and simultaneous quantification of the drug release using ^19^F MRI in response to a local temperature increase. The ^1^H CEST contrast enhancement in MRI can be switched on and off depending on the temperature because the CEST signal disappeared due to the release of intraliposomal water at the melting phase transition temperature of the liposomal membrane. In their study, the Z-spectra of liposomes containing the shift reagents were acquired at 25°C and 42°C.^19^ In contrast, we acquired the Z-spectra of the liposomes at 38°C and 44°C for consideration of the practical application of lipoCEST agents to theranostics.

Tagami et al. developed the HaT-liposome, composed of DPPC and Brij78 at a molar ratio of 96:4 and having an optimum formulation for contrast-enhanced MRI using Gd-DTPA and hyperthermia accompanied with drug release.^13^ In the present study, we used Brij72 instead of Brij78 as a surfactant for developing novel thermosensitive lipoCEST agents, because the melting point of Brij72 is slightly lower than that of Brij78 and the hydrophilicity–lipophilicity balance (HLB) number of Brij72 is smaller than that of Brij78.^13^ Tagami et al., however, reported that the use of Brij78 was superior to that of Brij72 in terms of thermosensitive release when DPPC and Brij72 or Brij78 was mixed at a molar ratio of 96:4. Although we attempted to acquire the NMR spectra of the lipoCEST agents comprising DPPC and Brij72 at a molar ratio of 96:4, the NMR spectral peaks were not observed. In contrast, they were observed in the lipoCEST agents comprised of DPPC and Brij72 at a molar ratio of 90:10. Thus, we selected this formulation in our study. We will attempt to use Brij78 instead of Brij72 and compare them as a further study in the near future.

As previously described, we took the RF pulse irradiation time for saturation as 1 s in this study. To investigate whether this irradiation time is sufficient for saturation, we performed two-dimensional exchange spectroscopy (2D EXSY) experiments (data not shown). In these experiments, the exchange phenomenon was sufficiently observed for a mixing time of 50 ms and we obtained the exchange rate of 566 Hz from the intra- to extraliposomal fractions. These results appear to indicate that the RF pulse irradiation time of 1 s is sufficient for saturation. Furthermore, we adopted 1 s for the RF pulse irradiation time to avoid the temperature rise of a sample.

Our results (Fig. 1) demonstrated that intra/extraliposomal water pools with distinct chemical shifts can be observed at temperatures below the melting phase transition for the thermosensitive lipoCEST agents developed in this study. The NMR spectra showed different chemical shifts between the intra- and extraliposomal water protons and different thermosensitivity depending on the agents (Fig. 1). In these agents, however, the NMR signal of the intraliposomal water protons was shifted from that of bulk water by 1 to 2 ppm at most. As shown in Fig. 1, the direction of the chemical shift was negative when Dy-DOTA-Na·3NaCl and Tb-DOTA-Na·3NaCl were used as shift reagents, whereas it was positive when Tm-DOTA-Na·3NaCl was used. In general, the direction of the chemical shift is determined by the magnetic anisotropy of the lanthanide complex, which is proportional to Bleaney’s constant.^7–9^ Bleaney’s constant characterizes each lanthanide ion. It is negative for Dy and Tb and positive for Tm.^7–9^ Thus, the results shown in Fig. 1 are consistent with those expected from the difference in the magnetic anisotropy. Furthermore, the chemical shift is proportional to the effective magnetic moment of the lanthanide complex and the concentration of shift reagents.^7–9^ As shown in Fig. 1, the NMR spectral peaks disappeared at 42°C. This appears to be due to the fact that the thermosensitive liposomes were disrupted and the intraliposomal agents were released at around 42°C, because the phase-transition temperature of the thermosensitive liposomes is approximately 42°C. To confirm that the finding shown in Fig. 1 is due to the release of the shift reagent, we measured the NMR spectra at 36°C again after heating the sample at 42°C and the signals from intraliposomal water protons were not observed for all lipoCEST samples, that is, the NMR spectra showed only the peak from bulk water protons (data not shown). This would be a convincing proof of the effective release of the shift reagent.

The chemical shift between the intra- and extraliposomal water protons is desired to be more than 10 ppm to saturate the intraliposomal water protons efficiently and to reduce interference with the magnetization transfer effect associated with endogenous proteins.^7,11^ as previously described. Therefore, we tried to shrink the thermosensitive lipoCEST agents (lipoA) through osmotic stress in order to enhance the BMS effect. As expected, the chemical shift increased to 4–6 ppm in the NMR spectra when the osmotic pressure was 1200 mOsm (Fig. 2). The direction of the chemical shift in the liposomes encapsulating Dy-DOTA-Na·3NaCl and Tb-DOTA-Na·3NaCl changed from negative to positive with increasing osmotic pressure (Fig. 2), which is probably due to the enhanced BMS effect. Note that the direction was unchanged (negative) in the liposome encapsulating Tm-DOTA-Na·3NaCl (Fig. 2). Furthermore, broadening and reduction of the signal intensity from the intraliposomal water protons were observed in all samples (Fig. 2). These findings are probably due to the shortening of the spin-spin relaxation time (T_2_), because the osmotic shrinkage of the liposome causes the release of the intraliposomal water and increases the concentration of the paramagnetic lanthanide metal, leading to T_2_ shortening.

As shown in Fig. 3, both the lipoA agents encapsulating Tm-DOTA-Na·3NaCl and Dy-DOTA-Na·3NaCl showed the desired temperature dependency of the chemical shift, that is, the NMR spectral peak disappeared at 42°C. This finding appears to indicate that the thermosensitivity of the lipoA agents is valid. However, the chemical shift of 4–6 ppm observed in the lipoA agent (Fig. 3) still appears to be insufficient for practical application of CEST MRI.

Terreno et al. demonstrated that the incorporation of amphiphilic Tm-based complexes into the composition of the non-spherical liposomal membrane can expand the chemical shift by the anisotropic orientation of liposomes to an external magnetic field.^9^ Therefore, we prepared novel thermosensitive lipoCEST agents denoted by lipoB encapsulating Dy-DOTA-Na with an osmotic pressure of 40 mOsm and incorporating Tm-DTPA-BSA as membrane constituents. As shown in Fig. 4, the chemical shift could be expanded to approximately 15 ppm by incorporating the Tm complex into the membrane composition, although the amount of the Tm complex did not affect the extent of the chemical shift (Fig. 4). Thus, we hypothesized that the direction of the chemical shift is more sensitive to the presence of Tm-DTPA-BSA than the value of the chemical shift. The direction of the chemical shift is dependent on the direction of the orientation of Tm-DTPA-BSA in liposomal membranes because the BMS effect largely depends on the angle between the principal axis of the rotation of Tm-DTPA-BSA and the direction of an external magnetic field.^7–9^ The value of the chemical shift is determined by the concentration of the lanthanide shift reagent, i.e., Dy-DOTA-Na, whereas Tm-DTPA-BSA would probably affect the direction of the chemical shift.

The NMR and CEST peaks were observed in the NMR- and Z-spectra obtained from the lipoB agent encapsulating Dy-DOTA-Na, respectively, at 36–38°C, whereas they disappeared at 42–44°C (Figs. 4, 5). Since the reduction in the CEST peak appears to reflect the quantity of the released agent, this finding suggests that the lipoB agent encapsulating Dy-DOTA-Na can be useful for monitoring drug release when applied to theranostics.

As previously described, two solutions were prepared for hydration in the present study: one is the solution containing the lanthanide complex with the counter ions, i.e., Ln-DOTA-Na·3NaCl, and the other is the solution without NaCl, i.e., Ln-DOTA-Na. When Ln-DOTA-Na·3NaCl with a concentration of 20 mM was used for hydration, the osmotic pressure became approximately 170 mOsm, and then a dialysis solution with an osmotic pressure of 1200 mOsm was required in order to obtain a larger chemical shift. However, when Ln-DOTA-Na with a concentration of 20 mM was used for hydration, the osmotic pressure became approximately 40 mOsm, accordingly the shrunken lipoCEST agents could be obtained under the isotonic stress (300 mOsm). Indeed, the final lipoB agent composing of DPPC and Brij72 at a molar ratio of 90:10 and incorporating 30 mol% of Tm-DTPA-BSA was dialyzed against NaCl solution with an osmotic pressure of 300 mOsm. This osmotic pressure is almost the same as that under physiological condition. In addition, the lipoB agent encapsulating only 20 mM Dy-DOTA-Na could shift the intraliposomal water proton peak by approximately 15 ppm from the bulk water one (Fig. 4). Although it appears that the lipoB agent can be applied to *in vivo* studies, the safety profiles of Ln-DOTA and Tm-DTPA-BSA need to be further studied in the future in order to apply the lipoB agent safely to humans.

To investigate whether Tm-DTPA-BSA affects the thermosensitivity of the lipoCEST agent, the temperature dependency of the calcein release from the agent was measured (Fig. 6). As is apparent from Fig. 6, the calcein release from the lipoCEST agent without Tm-DTPA-BSA significantly increased at 40°C, while it was stable at around body temperature (36°C). The lipoCEST agent containing 30 mol% of Tm-DTPA-BSA showed a similar temperature dependency to the lipoCEST agent without Tm-DTPA-BSA (Fig. 6). Although there were no significant differences in the leakage of calcein among the lipoCEST agents at temperatures lower than 36°C due to the large scatter of the data, the leakage in the lipoCEST agent containing 30 mol% of Tm-DTPA-BSA tended to be higher than that of the lipoCEST agent without Tm-DTPA-BSA or the lipoCEST agent containing 10 mol% of Tm-DTPA-BSA (Fig. 6). This finding might be due to the difference in stability of the membranes of the lipoCEST agents against temperature.

To further investigate the effect of Tm-DTPA-BSA, the stability of the thermosensitive lipoCEST agents was investigated by measuring the calcein retention as a function of incubation time at 36°C and 44°C (Fig. 7). As shown in Fig. 7A, although the scatter of the data was large, calcein was retained in all lipoCEST agents for at least 30 min at 36°C, suggesting that the stability of the lipoCEST agents is independent of the content of Tm-DTPA-BSA at lower temperature. It should be noted that although the calcein release in the lipoCEST agent containing 10 mol% of Tm-DTPA-BSA seems to go down from 10 to 20 min incubation time, there was no significant difference between them when analyzed by Student’s *t*-test. However, the leakage of approximately 2–10% was observed (Fig. 7A). The reason for this leakage is not clear, and thus further studies will be necessary to elucidate the reason for the leakage of calcein at low temperatures and to reduce this leakage for practical application. As shown in Fig. 7B, the percentage of calcein release in the lipoB agent incorporating 30 mol% of Tm-DTPA-BSA rapidly increased and eventually reached 33.9 ± 5.4% at 44°C after 30 min incubation. On the other hand, the calcein release from the lipoB agent incorporating 10 mol% of Tm-DTPA-BSA was limited to 13.0 ± 3.9% at 44°C (Fig. 7B). These results suggest that the lipoB agent incorporating 30 mol% of Tm-DTPA-BSA is more sensitive to temperature than that of the agent incorporating 10 mol% of Tm-DTPA-BSA at higher temperature. These findings are considered to be probably related to the stability of liposomal membranes caused by the molecular shapes of Tm-DTPA-BSA and Brij72. Brij72 used as a surfactant would make the liposomal membrane unstable due to its molecular shape with smaller polar head groups than the other membrane constituents.^20^ Tm-DTPA-BSA with large polar head groups would probably compensate the instability caused by Briji72 up to approximately 10 mol%. On the other hand, Tm-DTPA-BSA would make the liposomal membrane unstable by itself when its content is larger than 30 mol%. Accordingly, it appears that the lipoB agents incorporating 30 mol% of Tm-DTPA-BSA could release calcein more efficiently than that incorporating 10 mol% of Tm-DTPA-BSA at 40–44°C. Based on these findings, it is expected that the balance of contents between Tm-DTPA-BSA and Brij72 affects the stability of the liposomal membranes, especially at higher temperature.

Recently, it has been reported that some clinical trials have been performed to investigate the usefulness of ThermoDox^®^ (Celsion Corp., Lawrenceville, NJ, USA), which consists of the thermosensitive liposomes made with DPPC and doxorubicin.^21,22^ Furthermore, Tagami et al. have developed HaT-liposomes co-encapsulating Gd-DTPA and doxorubicin as previously described, and performed *in vivo* imaging studies on the drug distribution and delivery.^12^ When we consider the application of thermosensitive lipoCEST agents to theranostics, it is desired that these agents encapsulating drugs are stable at around body temperature, rapidly release the drugs under hyperthermic conditions (40–44°C), and have a chemical shift greater than approximately 10 ppm. It appears that the lipoB agent incorporating 30 mol% of Tm-DTPA-BSA as a membrane constituent and encapsulating Dy-DOTA-Na can satisfy these requirements, because it rapidly released calcein at 42–44°C but retained calcein at around 36°C (Fig. 6). In addition, the chemical shift of the intraliposomal water protons was approximately 15 ppm (Fig. 4). Thus, this agent appears to be the most appropriate as a themosensitive lipoCEST agent for application to theranostics.

## Conclusion

In the present study, we successfully developed novel thermosensitive lipoCEST agents and characterized them. We also demonstrated that a large chemical shift derived from intraliposomal water protons is obtained by shrinking spherical liposomes through osmotic stress and that the chemical shift value in the lipoCEST agent containing both Dy-DOTA-Na and Tm-DTPA-BSA (lipoB) is much larger than that in the lipoCEST agent containing only Ln-DOTA-Na·3NaCl (lipoA). Our preliminary results suggest that our thermosensitive lipoCEST agents are useful and can be applied to theranostics. However, the present study is limited to *in vitro* experiments. Therefore, we will perform *in vivo* studies in the near future in order to investigate the feasibility of the practical application of the thermosensitive lipoCEST agents developed in this study.
